# Drought-induced delays in stem hydraulic development shape gas exchange and growth recovery in Douglas fir

**DOI:** 10.1093/plphys/kiaf654

**Published:** 2025-12-17

**Authors:** Franklin Alongi, Timo Knüver, Scott A M McAdam, Yanick Ziegler, Andreas Gast, Nadine K Ruehr

**Affiliations:** Karlsruhe Institute of Technology (KIT), Institute of Meteorology and Climate Research—Atmospheric Environmental Research (IMKIFU), Garmisch-Partenkirchen 82467, Germany; Karlsruhe Institute of Technology (KIT), Institute of Meteorology and Climate Research—Atmospheric Environmental Research (IMKIFU), Garmisch-Partenkirchen 82467, Germany; Purdue Center for Plant Biology, Department of Botany, Department of Botany and Plant Pathology, Purdue University, West Lafayette, IN 47907, United States; Karlsruhe Institute of Technology (KIT), Institute of Meteorology and Climate Research—Atmospheric Environmental Research (IMKIFU), Garmisch-Partenkirchen 82467, Germany; Karlsruhe Institute of Technology (KIT), Institute of Meteorology and Climate Research—Atmospheric Environmental Research (IMKIFU), Garmisch-Partenkirchen 82467, Germany; Karlsruhe Institute of Technology (KIT), Institute of Meteorology and Climate Research—Atmospheric Environmental Research (IMKIFU), Garmisch-Partenkirchen 82467, Germany; Karlsruhe Institute of Technology (KIT), Institute of Geography and Geoecology (IfGG), Karlsruhe 76131, Germany

## Abstract

The limiting factors of tree recovery from drought, particularly the coordination between carbon sources and sinks, remain poorly understood. In this study, juvenile Douglas fir (*Pseudotsuga menziesii*) were exposed to 28 d of mild or severe drought, followed by 35 d of recovery. We continuously monitored CO₂ and H₂O fluxes in shoots and roots to derive gas exchange and carbon accumulation, while measuring basal area to estimate stem growth and sapwood development. To identify underlying mechanisms of drought recovery, we periodically measured nonstructural carbohydrates (NSC), midday water potential (Ψ_md_), and foliar abscisic acid (ABA). We found no evidence that ABA or Ψ_md_ limited gas exchange recovery, with stomatal conductance recovery instead related to drought-induced reductions in sapwood development. While carbon accumulation ultimately recovered to control levels following mild stress, severe stress caused persistent impairments, ultimately reducing carbon accumulation by 51%, with stem growth similarly affected. We found no evidence of growth being limited by NSC, which remained abundant. However, we suggest that drought-induced limitations to stem development govern this pattern. This became clear when considering the diurnal growth cycle, where daytime growth was largely absent in trees after exposure to severe drought despite accounting for up to 30% of total growth in control trees. Daytime growth appeared to depend on sufficient sapwood area, which likely buffered xylem tension to support growth conditions. Our findings suggest drought-induced reductions of stem hydraulic development constrain the recovery of gas exchange and growth. Further, altered diurnal growth patterns may explain prolonged productivity declines in forests following drought.

## Teaser

Drought delays stem water transport development in Douglas fir, constraining recovery of photosynthesis and growth by disrupting daytime expansion patterns.

## Introduction

Forests are major carbon (C) sinks, but their capacity to accumulate C is increasingly threatened by the growing intensity of drought events (Liu et al. 2024). Severe drought events impose physiological constraints that alter C source and sink coordination, leading to delayed or limited recovery (Ruehr et al. 2019). While impaired forest productivity following drought is well documented in observational field studies (Serra-Maluquer et al. 2018), these studies struggle to identify the physiological mechanisms limiting recovery. In contrast, experimental studies can isolate physiological responses to drought, but few examine recovery—leaving a critical gap in understanding how impairments such as reduced photosynthesis, nonstructural carbohydrate (NSC) reserves, and tissue damage persist beyond drought and affect long-term carbon accumulation. Physiological impairment and damage are known to scale with drought intensity (Ruehr et al. 2019), but the extent to which drought-induced damage affects tree recovery potential is still largely unexplored. Understanding how drought severity mechanistically alters tree resilience during recovery is essential, as it directly affects long-term C storage efficiency and ultimately the role of forests as C sinks.

Effective stomatal regulation is crucial for minimizing water loss and maintaining hydraulic function during drought so that hydraulic impairment does not delay recovery. Stomatal conductance (g_sw_) is tightly regulated by tree water status, with stomatal closure initiated by mild water stress and further suppressed under severe drought by elevated foliar abscisic acid (ABA) (McAdam and Brodribb 2014). Persistent reductions in g_sw_ during drought recovery have been attributed to delayed hydraulic restoration, assessed through measures of water potential (ψ), as well as continued signaling from ABA (Brodribb and McAdam 2013; Rehschuh et al. 2020). However, it is important to distinguish that ψ reflects the plant water status as indicated by xylem tension, whereas overall hydraulic conductance also depends on the extent of functional xylem present in the sapwood. The sapwood determines the tree's capacity to transport water to the canopy and thus, its potential for recovering stomatal conductance (Matthaeus et al. 2022). Metrics such as the ratio of sapwood area to leaf area (Huber value, H_v_) assess the hydraulic supply–demand balance. H_v_ values are closely linked to stem hydraulic conductance across species (Mencuccini et al. 2019), with higher H_v_ associated with greater g_sw_, as the hydraulic system is better equipped to maintain guard cell turgor (Zlobin et al. 2024). Due to the formation of embolized xylem conduits during severe drought stress, g_sw_ recovery is likely dependent on new xylem formation. As a result, an increase in H_v_ may be necessary to restore hydraulic capacity, enabling trees to recover g_sw_ and ultimately photosynthetic function.

Reduced g_sw_ limits CO_2_ uptake and photosynthetic assimilation (A_net_), restricting C availability for secondary metabolism and leading to shifts in C allocation. These shifts often reduce respiration and minimize growth, ultimately conserving C for primary metabolic function (Rodríguez-Calcerrada et al. 2021). Furthermore, slight reductions in water potential under drought stress can limit growth by imposing turgor constraints on cellular expansion and differentiation (Körner 2015; Peters et al. 2021), indicating a sink-driven control of growth during drought. As such, turgor-sensitive “resting” sink tissues can simultaneously experience an accumulation of carbohydrates and a reduced input from recent assimilates during drought (Hagedorn et al. 2016), assuming C input remains above respiratory demand. Therefore, drought-induced carbohydrate accumulation in sink tissues may lead to feedback inhibition of photosynthesis (Paul and Foyer 2001; Tian et al. 2024), decoupling the limitations of photosynthesis from stomatal control. Thus, the noninstantaneous recovery of A_net_, and ultimately carbon uptake and growth is not readily attributed to either persistent hydraulic impairment or metabolic inhibition alone, highlighting the challenge of disentangling their relative contributions in limiting post-drought recovery.

Physiological damage sustained during drought stress may increase the C cost of recovery due to the need to repair or replace damaged tissues (Ruehr et al. 2019), ultimately delaying the resumption of growth. Cellular and hydraulic damage sustained under severe drought stress can directly impair the post-drought recovery of gas exchange (Rehschuh et al. 2020). This may be particularly relevant in conifers, which tend to maintain higher hydraulic safety margins and are therefore more embolism avoiding than angiosperm forest species (Johnson et al. 2011). Additionally, conifers have comparatively little parenchyma in their woody tissues compared with angiosperms (Morris et al. 2016), which store NSC that could aid in embolism refilling. However, whether embolism dissolution naturally occurs at all following drought-induced cavitation remains disputed (Choat et al. 2019). As such, conifers likely rely on the formation of new xylem tracheids via new sapwood area to overcome losses of hydraulic function sustained during drought stress, constituting a high C cost during recovery (Brodribb and Cochard 2009). This high C demand of tissue replacement and repair following stress release may be why tree growth recovery often takes longer than the recovery of gas exchange (Kannenberg et al. 2022) and could explain why the lag in growth recovery in conifers directly scales with the intensity of drought (Sergent et al. 2014). However, drought severity-induced shifts in tree C allocation during stress recovery are poorly understood due to difficulties in assessing whole-tree C status. This knowledge gap complicates our ability to assess C costs associated with drought stress and how these ultimately translate to long-term reductions in C accumulation and growth ability.

NSCs represent a major C sink in conifers and play a critical role in sustaining tree function during drought stress. As drought intensifies and photosynthesis (A_net_) declines, stored NSC are mobilized to support cellular respiration and lower osmotic potential (Long and Adams 2023). NSC depletion during drought is more frequent in gymnosperms than in angiosperms and is closely related to xylem hydraulic vulnerability (Adams et al. 2017). Similarly, sustaining high NSC levels during drought is linked to increased drought survival in conifers (Garcia-Forner et al. 2016), suggesting that NSC retention may be prioritized over other metabolic processes like growth (Stefaniak et al. 2024). However, whether NSC accumulation is actively or passively regulated during drought remains widely debated, as growth typically ceases before photosynthesis due to turgor limitations on cell expansion, which could passively facilitate NSC accumulation for as long as C assimilation remains above respiratory demand (Hartmann and Trumbore 2016).

During post-drought recovery, NSC influences the ability of trees to regain optimal function and growth. In *Picea abies* it has been observed that NSCs in xylem parenchyma that were maintained during drought stress were depleted 1 wk following recovery, which corresponded to increased xylem conductance (Tomasella et al. 2017). Similarly, accumulated NSCs during drought are linked to the rapid xylem cell formation upon recovery (Martínez-Sancho et al. 2022), allowing trees to develop functional new xylem to compensate for nonfunctional, embolized xylem. Due to the critical role of NSCs in tree survival during drought and in supporting recovery, prioritization of NSC maintenance may limit long-term growth (Kannenberg and Phillips 2020; Guo et al. 2021). Determining the prioritization between NSC maintenance and growth requires an understanding of whole-tree C balance during both drought and recovery; however, few studies integrate NSC responses to stress with whole-tree C exchange measurements (Hartmann et al. 2015; Hartmann and Trumbore 2016). Simultaneous measurements of gas exchange and NSC dynamics can clarify how trees allocate between storage and growth following stress-induced changes in carbon balance, providing critical insights into the physiological mechanisms that govern stress recovery and long-term forest resilience.

In this study, we aimed to identify how drought severity mechanistically limits the recovery of key physiological processes related to C acquisition and allocation in Douglas fir (*Pseudotsuga menziesii*) seedlings. This study builds on existing conceptual frameworks that describe the dual impacts of drought severity on hydraulic function and metabolic C dynamics during recovery (Ruehr et al. 2019). Douglas fir was selected because of its widespread distribution across North America, as well as its increasing role in European forestry due to its higher drought tolerance and growth resilience than many native species (Stangler et al. 2022). Specifically, this study uses whole-tree gas flux chambers to examine the effects of mild and severe drought on C assimilation, accumulation, and growth during recovery, while foliar ABA and branch NSC concentrations were periodically sampled to identify phytohormone regulation and C reserve status. We addressed the following hypotheses:

1. Foliar ABA and Ψ_md_ will increasingly limit gas exchange during drought and initial post-drought recovery.
2. C assimilation and C accumulation will acclimate to mild stress and quickly recover, while severe drought stress will more greatly limit recovery.
3. NSC will accumulate following growth cessation and ultimately decrease during severe stress (due to higher respiration relative to photosynthesis), while stem growth will partially recover only after NSC reserves are restored.

## Results

### Dynamics of water potential, abscisic acid, and stomatal conductance

**Figure 1.**
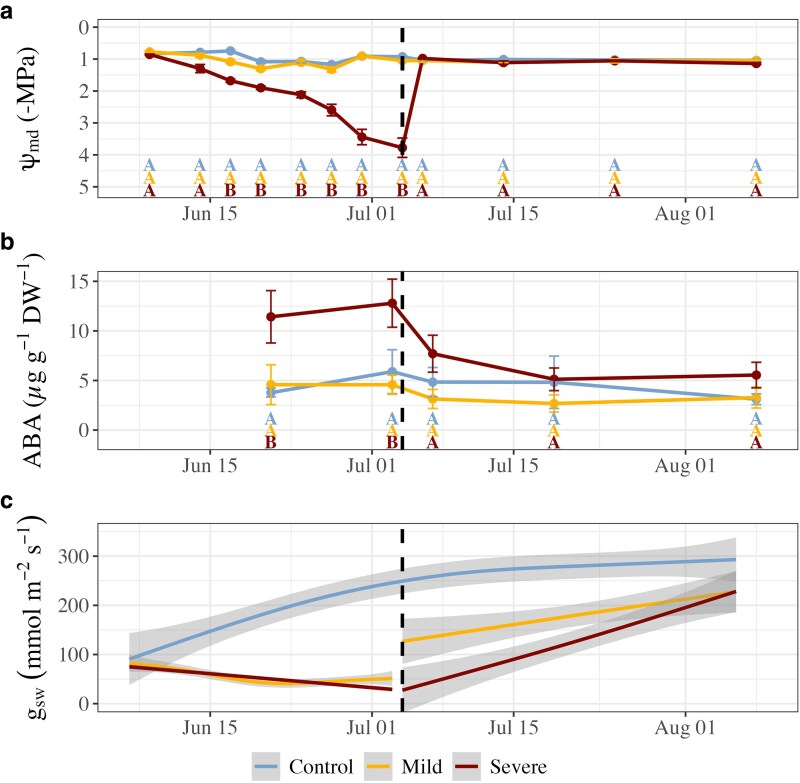
Dynamics of water potential, abscisic acid levels, and stomatal conductance during drought and recovery in Douglas fir juveniles. Midday branch water potential (a, Ψ_md_) was measured periodically on pilot trees (*n* = 5 per treatment). Foliar abscisic acid (b, ABA) concentrations were measured periodically in pilot trees (*n* = 5 to 7 per treatment). Error bars are ±SE between individual mean values in panels a and b. Uppercase letters in panels a and b indicate significant pairwise differences at each timepoint determined post hoc using Tukey's Honest Significant Difference. Mean daytime (09.00 to 14.00) stomatal conductance (g_sw_, c) rates for are reported for chamber seedlings (*n* = 4 to 6 per treatment). Generalized additive models were fit to produce smoothing lines for panel c, with shaded gray are representing ±SE. The vertical black dashed line in all panels indicates the transition from drought to recovery.

Midday water potential (ψ_md_) in the mild drought treatment plants remained close to or at control levels (−1.0 ± 0.1 MPa) throughout the drought period (Fig. 1a), despite lower volumetric soil water content (SWC). In the severe treatment plants, ψ_md_ progressively decreased throughout the drought period. The severe drought treatment plants reached −3.8 ± 0.3 MPa by the end of drought stress, corresponding to 70% to 85% loss of conductivity for this species (Chauvin et al. 2019). Following the release of drought stress, ψ_md_ in the severe treatment plants returned to levels of the control treatment plants within 2 d.

Foliar ABA levels in the severe drought treatment plants more than doubled by Day 14 of drought stress (*P* = 0.016, *t*_12_ = −2.801, Fig. 1b) and remained high through the end of drought (*P* = 0.024, *t*_12_ = 2.467), corresponding to a strong decrease in ψ_md_. We observed no differences in ABA levels between the control and mild drought treatment plants during either drought or recovery, corresponding to the lack of a detectable difference in ψ_md_. Within 2 d of recovery, ABA levels declined in the severe treatment to levels measured in control treatment plants as ψ_md_ recovered and were maintained throughout the recovery period.

**Figure 2.**
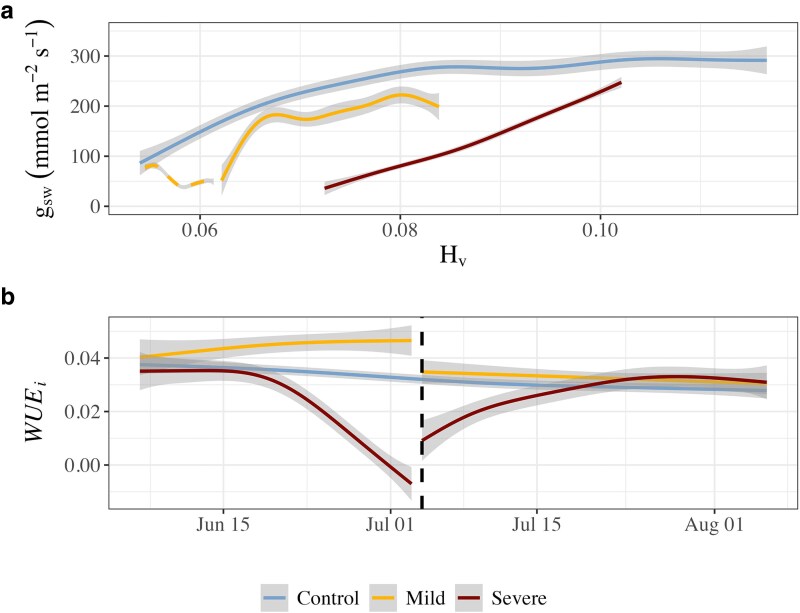
Development of daytime g_sw_ with Huber value (sapwood area: leaf area; H_v_) and WUE_i_ throughout the experimental period. Panel a displays the relation of g_sw_ with H_v_ (*n* = 4 to 6), with the drought period for the mild treatment denoted by a dashed line, while it is omitted for the severe treatment due to observed effects of abscisic acid on g_sw_, as well as stem contraction due to dehydration. Panel b displays the time series of daytime (09.00 to 14.00) WUE*_i_* (*n* = 4 to 6). The vertical dashed black line indicates the transition from drought to recovery. Generalized additive models are fit to produce smoothing lines in both panels, with the shaded gray area representing ±SE.

**Figure 3.**
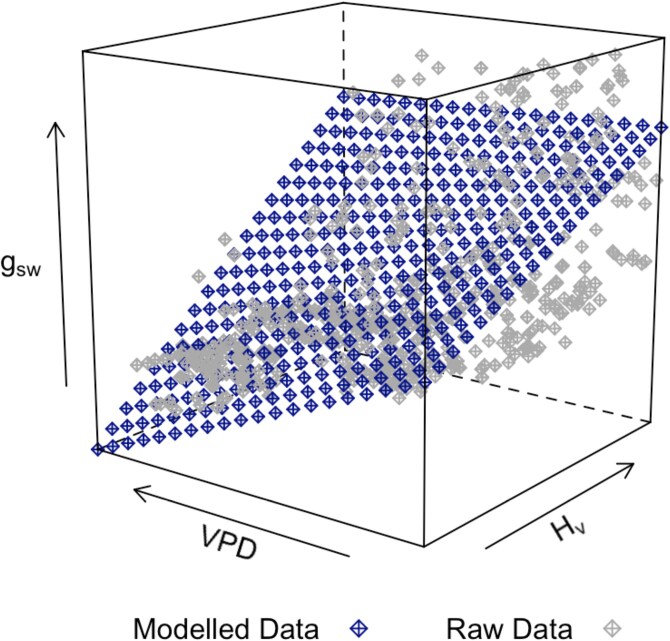
3D representation of the influence of vapor pressure deficit (VPD) and Huber value (sapwood area: leaf area; H_v_) on stomatal conductance (g_sw_). The direction of increase for each variable is indicated with an arrow. Gray points represent the raw data, while the blue surface represents model-predicted values from a linear mixed-effects model. The model included additive and interactive effects of VPD and Hv, along with treatment as a fixed effect, and plant identity (PlantID) as a random intercept to account for repeated measures. Model predictions were generated by creating a regular grid of H_v_ and VPD values across their measured range. The predict() function was used to compute predicted g_sw_ values across this grid. Raw and modeled data were merged and visualized using the cloud() function from the *lattice* package, with symbol shapes and colors differentiating observed and predicted data. This modeling approach was exploratory and aimed to assess potential interactions between structural and environmental controls on stomatal conductance, not to produce causal inference.

Stomatal conductance (g_sw_) responded according to drought severity (Fig. 1c). Mild stress plants moderately decreased g_sw_ values by the end of drought stress to 45.02 ± 7.11 mmol m^−2^ s^−1^, which was 81% lower than the control treatment (*P* = 0.004, *t*_12_ = −3.526), as the control treatment plants instead continuously increased g_sw_. This increase in g_sw_ in control treatment plants over the course of the experiment was strongly related to increases in Huber value (stem sapwood:leaf area; H_v_), suggesting increases in hydraulic supply occurred due to continual stem growth (Fig. 2a), until a H_v_ of ∼0.08 was reached, at which point maximal g_sw_ was sustained. In individuals exposed to severe stress, g_sw_ consistently decreased throughout the drought period, reaching 31.52 ± 2.35 mmol m^−2^ s^−1^, or 86% lower than the control by the end of drought (*P* = 0.004, *t*_12_ = −3.547). An exploratory model to determine the contributing factors to g_sw_ during the experimental period revealed that g_sw_ was negatively related to VPD (*P* < 0.001, *t*_904.64_ = −9.03), with the effect of VPD weakened by increasing H_v_ (*P* < 0.001, *t*_908.81_ = 5.25; Fig. 3). The highest VPD was recorded in the severe treatment at the end of the drought period (∼2.4 ± 0.2 kPa), while the VPD in the control treatment remained consistent (∼1.5 ± 0.1 kPa, Fig. S1d).

Within 2 d of recovery, the mild stress treatment immediately increased g_sw_ to 90.42 ± 19.13 mmol m^−2^ s^−1^, which was above prestress levels. g_sw_ continued to recover in the mild stress treatment, corresponding to increases in H_v_ (Fig. 2a), ultimately reaching 204.9 ± 67.3 mmol m^−2^ s^−1^, 30% below that of the control (n.s. due to high SE). Interestingly, this g_sw_ was associated with a H_v_ of ∼0.08, a similar value to that which facilitated the highest g_sw_ in the control group (Fig. 2a). In contrast, there was no immediate recovery of g_sw_ following severe stress despite an immediate reduction in foliar ABA, with prestress levels not reached until 2 d into recovery. Despite this, g_sw_ ultimately recovered to similar levels as the mild stress treatment, reaching 234.6 ± 119.2 mmol m^−2^ s^−1^ by the end of recovery, or 20% below that of the control (n.s. due to high SE). Transpiration (E) was lower than the control in both stress treatments; however, a higher E was observed in the severe treatment than the mild treatment during both drought and recovery (Fig. S2), likely due to the generally higher VPD in the severe treatment chambers (Fig. S1d).

### Carbon assimilation and accumulation

**Figure 4.**
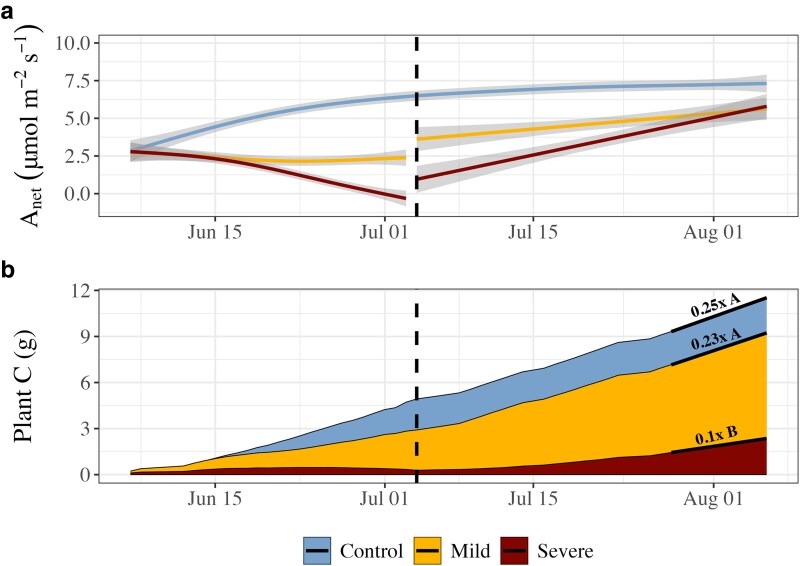
Net carbon (C) assimilation and accumulation from Douglas fir seedlings in gas exchange chambers. Mean daytime (09.00 to 14.00) net photosynthesis rates (a, A_net_) for each individual tree were fit with generalized additive models to produce smoothing lines, with the shaded gray area are representing ±SE (*n* = 4 to 6). Carbon accumulation is reported in grams of carbon from chamber seedlings (b). Data reflect daily sums of CO_2_ gas exchange of shoot and root sections indicating the whole-seedling net carbon accumulation over the course of the experiment. In panel b, linear models are fit for the final 10 d of recovery for each treatment (black lines) with the slope reported above each line to indicate daily C accumulation rates (*n* = 4 to 6). Significant differences of the slopes (uppercase letters) were calculated using Tukey's Honest Significant Difference. The dashed black line indicates the transition from drought to recovery in both panels.

Net photosynthetic assimilation (A_net_) largely mirrored drought intensity throughout the experimental period (Fig. 4a). During the experimental drought, control plants continuously increased daytime A_net_ in coordination with g_sw_, reaching 6.56 ± 0.74 µmol m^−2^ s^−1^ by the end of drought stress. A_net_ in the mild drought treatment plants fluctuated around initial values, with an A_net_ of 2.25 ± 0.55 µmol m^−2^ s^−1^ by the end of drought, 66% below that of the control (*P* < 0.001, *t*_12_ = −5.174). As a result, intrinsic water use efficiency (WUE_i_) remained higher than the control during mild stress (Fig. 2b). WUE_i_ during severe stress ultimately decreased below control levels and turned negative due to net daytime respiration (Fig. 4b), suggesting metabolic and nonstomatal limitations to photosynthesis.

As with g_sw_, A_net_ in the mild stress treatment immediately recovered to higher rates than prestress and continued to increase through recovery in-line with increases in H_v_, ultimately reaching an A_net_ of 5.53 ± 1.25 µmol m^−2^ s^−1^ at the end of recovery, 26% less than the control on average (n.s.). As a result, WUE_i_ in the mild stress trees immediately returned to control levels upon recovery (Fig. 2b), reflecting the release of stomatal limitations to photosynthesis. A_net_ in the severe stress treatment immediately increased upon recovery; however, unlike the mild stress treatment, remained below predrought levels for ∼10 d, 5 d longer than g_sw_. This corresponded to WUE_i_ temporarily remaining below control levels during the first 2 wk of recovery. As with g_sw_, A_net_ ultimately recovered to similar levels following severe stress as after mild stress, approximately 26% below control levels on average (n.s.). This delayed recovery of both A_net_ and g_sw_ was associated with a higher H_v_, suggesting that new basal area was necessary to regain the hydraulic supply required to facilitate maximal gas exchange. In support of this, the intercorrelations between g_sw_, A_Net,_ and H_v_ were strongest in the mild and severe treatments (Table S1), as the control treatment reached sufficient H_v_ to support maximal gas exchange earlier in the season (Fig. 2a).

Net C accumulation was limited by drought stress during both the drought and recovery period. While control plants accumulated 4.93 ± 0.88 gC during the drought period, accumulated C was on average 41% lower under mild stress (*P*  *=* 0.040, *t*_12_ = −1.92) and 94% lower under severe stress (*P* = 0.001, *t*_12_ = −4.07). These trends were similar in both above- and belowground compartments (Fig. S3), with both aboveground net C assimilation and belowground respiration rates decreasing with drought intensity (Table 1, Fig. S4). During the recovery period, control trees accumulated an additional 6.61 ± 1.31 gC, with a C accumulation rate of 0.25 ± 0.01 g C d^−1^ during the final week of recovery, similar to the mild treatment (*P*  *=* 0.76). The severe stress treatment plants accumulated 69% less carbon than the control treatment plants during recovery (*P* = 0.011, *t*_11_ = −3.075). Daily C accumulation rates at the end of recovery were similar between the control and mild treatment but were 51% lower in the severe treatment (Fig. 4b, Tukey HSD).

**Table 1.** Net carbon accumulation in grams per day (g C d^−1^) across chamber compartment and experimental period.

Compartment	Drought treatment	Drought period (g C d^−1^)	Recovery period (g C d^−1^)	Experimental period (g C d^−1^)
Shoot	Control	0.24 ± 0.04 **A**	0.27 ± 0.04 **A**	0.27 ± 0.04 **A**
	Mild	0.14 ± 0.04 **A**	0.24 ± 0.06 **A**	0.19 ± 0.05 **A**
	Severe	0.03 ± 0.01 **B**	0.10 ± 0.04 **B**	0.07 ± 0.03 **B**
Root	Control	−0.06 ± 0.01 **A**	−0.09 ± 0.01 **A**	−0.07 ± 0.01 **A**
	Mild	−0.04 ± 0.01 **AB**	−0.06 ± 0.02 **A**	−0.04 ± 0.02 **AB**
	Severe	−0.02 ± 0.00 **B**	−0.04 ± 0.01 **B**	−0.03 ± 0.01 **B**
Plant	Control	0.18 ± 0.03 **A**	0.19 ± 0.04 **A**	0.18 ± 0.03 **A**
	Mild	0.10 ± 0.03 **B**	0.18 ± 0.05 **A**	0.15 ± 0.04 **A**
	Severe	0.01 ± 0.01 **C**	0.06 ± 0.03 **B**	0.04 ± 0.02 **B**

Reported are treatment means for each compartment with standard error. Bold letters indicate significant differences calculated using Tukey's Honest Significant Difference. C loss (net respiration) is indicated by negative numbers, while C gain (net assimilation) is indicated by positive numbers.

### Nonstructural carbohydrates

**Figure 5.**
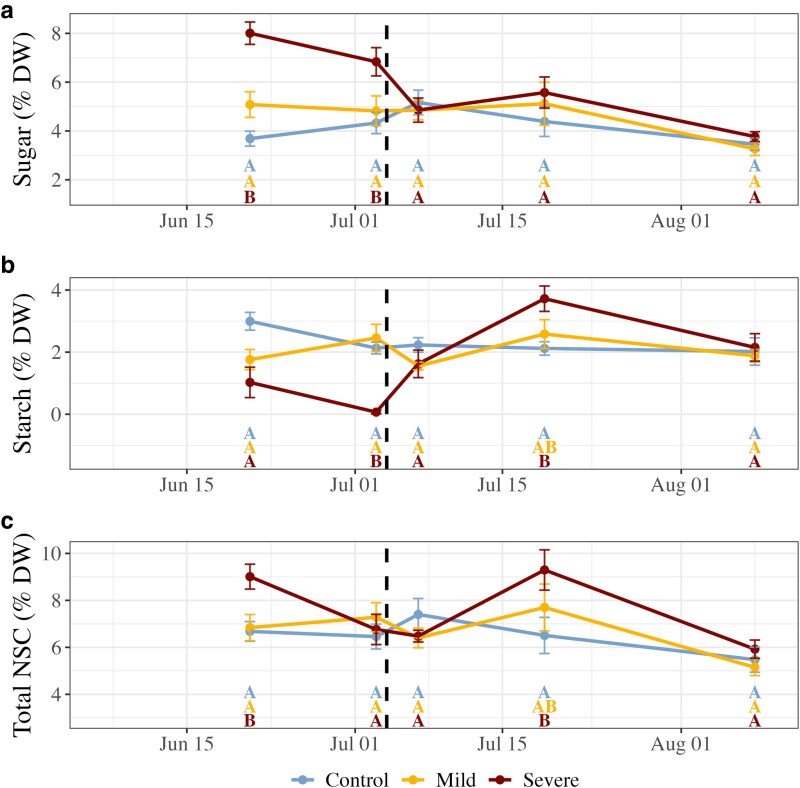
NSC concentrations in axial branch tissues of Douglas fir pilot seedlings (*n* = 5 to 7 per drought treatment). Displayed are free sugar (a, glucose + fructose + sucrose), starch (b) and total NSC (c, free sugar + starch). Error bars indicate standard error, while the dashed black line in all panels indicates the transition from drought to recovery. Uppercase letters indicate significant pairwise differences per timepoint determined post hoc using Tukey's Honest Significant Difference.

Branch free sugar concentrations were highly dynamic during the drought period (Fig. 5a), more than doubling under severe drought stress to 8.01 ± 0.49% DW after 14 d compared with the control (*P* < 0.001, *t*_12_ = 6.942). By the end of the drought period (28 d), free sugar concentrations were still 58% greater than the control treatment plants (*P* = 0.004, *t*_18_ = 2.962). In mild stress treatment plants, axial branch free sugar concentration remained unchanged throughout the drought. Within 2 d of recovery, sugar concentrations in the severe treatment individuals returned to concentrations measured in control treatment plants and did not vary at any timepoint in the 35 d recovery.

Axial branch starch concentrations responded inversely to free sugar during drought stress (Fig. 5b). By 14 d into drought stress, starch decreased by 70% to 1.03 ± 0.49% DW in the severe treatment individuals and was no longer detectable by the end of drought. As was the case with free sugars, starch concentrations did not differ at any timepoint between the mild drought treatment plants and the control treatment plants. Within 2 d of recovery, starch concentrations in the severe drought treatment plants quickly increased to levels measured in control treatment plants and were higher than the control 15 d into recovery (*P* = 0.015, *t*_17_ = 2.689). Starch concentrations ultimately returned to levels measured in control treatment plants by the end of the 35 d recovery.

Total NSCs (sum of free sugar and starch) differed between treatments only at the mid-drought timepoint (Fig. 5c), where total NSC reached 9.03 ± 0.52% DW, or was 28% higher in the severe drought-stressed plants than the control treatment plants (*P* = 0.041, *t*_12_ = 2.289). Of this, about 90% of NSC was allocated in free sugars. Total NSC no longer differed by treatment by the end of drought and did not differ at any time during recovery.

### Growth and biomass accumulation

**Figure 6.**
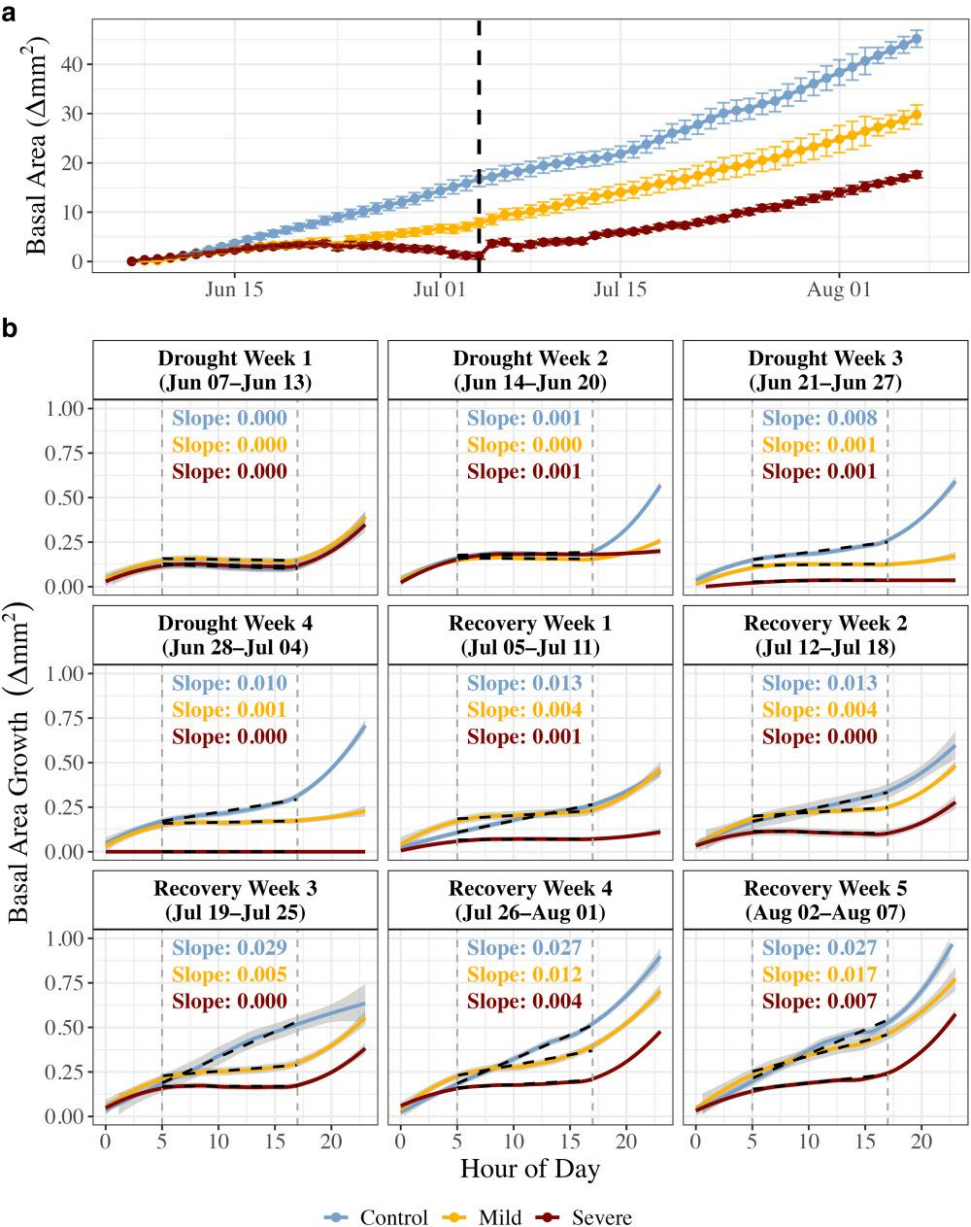
Basal area and growth metrics of Douglas fir chamber seedlings during the experimental (drought and recovery) period. Displayed are daily changes in stem basal area (a, *n* = 4 to 6 per treatment), with error bars indicating standard error and the dashed black line indicating the transition from drought to recovery. Diurnal growth dynamics during each week of the experimental period are reported with growth calculated following the zero-growth concept (b), whereby no growth is assumed during periods of stem dehydration. The shaded error indicates standard error. Linear regression lines and the corresponding slopes represent the midday growth rate (between 5.00 and 17.00).

Stem basal growth proportionally reflected drought intensity throughout the drought and recovery period (Fig. 6a). While control plants increased basal area on average by 0.58 ± 0.05 mm^2^ d^−1^ during drought, growth decreased by 47% under mild stress to 0.28 ± 0.03 mm^2^ d^−1^ (*P* = 0.038, *t*_12_ = −1.95). In the severe drought treatment plants, basal area growth ultimately stopped and began shrinking due to stem dehydration (Fig. 6a), with an average growth rate of only 0.04 ± 0.02 mm^2^ d^−1^. Basal area growth in the mild stress treatment immediately accelerated upon rewatering; however, it was on average 25% (0.61 ± 0.06 mm^2^ d^−1^) than the control (0.80 ± 0.05 mm^2^ d^−1^), although not statistically different (*P* = 0.351). Following rewatering, severely stressed plants quickly regained their previous maximum diameter; however, further growth (and therefore stem hydraulic development) did not resume until the second week of recovery (Fig. 6b). Basal area expansion rates remained below the control, leading to a 42% lower stem growth on average during recovery (0.40 ± 0.02 mm^2^ d^−1^) than the control treatment plants, though not statistically significant (*P* = 0.085, *t*_12_ = −1.88).

Notably, differential growth patterns were apparent on a diurnal scale, whereby daytime basal area growth appeared in the control trees by the third week of the drought period, while growth remained restricted to nighttime in the mild and severe treatments throughout drought (Fig. 6b). Daytime growth appeared in the mild treatment by the third week of recovery and by the final week of recovery in the severe treatment. An exploratory model to determine the contributing factors to daytime growth revealed daytime growth rate was positively related to H_v_ at the start of the day (*P* = 0.001, *t_682.06_* = 2.62) and negatively related to VPD (*P* = 0.001, *t_290.33_* = −2.28; Fig. S5). Net daytime growth appeared possible once trees reached an H_v_ of ∼0.08 or greater in mild and control trees but occurred at a slightly higher H_v_ in the severe treatment (∼0.10).

Root-to-shoot ratios revealed drought-dependent carbon distribution between biomass compartments (Figure S6). The root:shoot ratio was highest in control plants (0.67 ± 0.04) despite having the largest diameter expansion, reflecting elevated allocation to belowground tissues. The root:shoot ratios of mild and severe treatment plants were both below that of the control (mild: 0.42 ± 0.06, *P*  *=* 0.001, *t*_13_ = −4.134; severe: 0.37 ± 0.06, *P* < 0.001, *t*_13_ = −4.323), indicating lower allocation to belowground tissues relative to aboveground tissues, in line with reduced belowground respiration (Fig. S4a). A summary of biomass between belowground woody biomass, aboveground woody biomass, and needle tissues can be found in Table S2.

## Discussion

### Drought severity-induced hydraulic and ABA control of stomatal conductance

We found that drought stress impacted g_sw_ in an intensity-dependent manner; however, this was not solely attributable to Ψ_md_ or phytohormone status. Stomatal closure during drought is regulated both passively and actively in gymnosperms, whereby water stress passively decreases guard cell turgor early in drought, while active accumulation of ABA further reduces g_sw_ (McAdam and Brodribb 2014; Tombesi et al. 2015; Manandhar et al. 2024). In agreement, branch Ψ_md_ gradually decreased, triggering an increase in foliar ABA levels as the severe drought progressed, corresponding to gradual and near complete stomatal closure in these plants (Fig. 1c). Under mild drought stress, g_sw_ remained around initial values while Ψ_md_ and ABA levels were maintained. While this could be interpreted as g_sw_ not responding to the mild drought treatment, the control individuals contrastingly increased g_sw_ over the same time period. Considering leaf-out was complete by the start of the drought period, we attribute the increasing g_sw_ in the control treatment plants to greater hydraulic supply from sapwood growth, indicating that g_sw_ was hydraulically limited in all treatments during the early growing season by inadequate stem hydraulic supply (Song et al. 2024). This is supported by the positive relationship between H_v_ (stem sapwood area: leaf area) and g_sw_ (Fig. 2a), with H_v_ and chamber VPD identified as key factors contributing to g_sw_ throughout the experimental period (Fig. 3). Thus, the lack of g_sw_ increase in the mild stress treatment plants may be due to minimal sapwood growth during the drought treatment, suggesting that g_sw_ remained low due to stem hydraulic supply. This effect of stem hydraulic supply, however, was not reflected in our measurements of Ψ_md_. It is important to distinguish that Ψ_md_ represents the plant water status, ie, xylem tension, whereas hydraulic supply refers to the xylem water transport capacity, which closely depends on the functional sapwood area. Diurnal traces of whole plant gas exchange and water potential in conifers during a drought have demonstrated that the critical water potential thresholds conferring reductions in gas exchange can be subtle given the passive regulation of stomatal control in these species under mild drought stress (McAdam and Brodribb 2014; Manandhar et al. 2024). Thus, small yet physiologically relevant differences in water potential likely existed between the mild and control treatments but appeared to be below our detection threshold or were present outside of our sampling timeframe.

After rewatering, we observed an immediate partial recovery of g_sw_ in mild-stressed plants and a gradual increase in g_sw_ in the severely drought-stressed plants. While high levels of ABA contributed to greatly reducing g_sw_ during severe stress, ABA recovered to control levels within 2 d of recovery (Fig. 1b). Accumulation of foliar ABA has been linked to delayed g_sw_ recovery on rewatering in *Cinnamomum camphora* and in conifers such as *Pinus radiata* (Brodribb and McAdam 2013; Duan et al. 2020); however, this was not observed in *Pinus sylvestris* (Zlobin et al. 2023), nor in field studies (Skelton et al. 2017). While drought-induced xylem embolism can limit g_sw_ recovery in conifers (Brodribb and Cochard 2009; Rehschuh et al. 2020), we found Ψ_md_ immediately returned to control values following rewatering (Fig. 1a), despite Ψ_md_ reaching ∼−3.8 MPa during severe stress, corresponding to an estimated loss of conductivity between 70% and 85% (Chauvin et al. 2019). This indicates that the recovery of water status (Ψ_md_) occurred rapidly, while full hydraulic recovery was more gradual and likely dependent on new xylem formation. The gradual increase of g_sw_ in the drought-treated plants during recovery was likely related to drought-induced limits of stem hydraulic supply (estimated via H_v_) and embolized conduits both being relieved by new sapwood growth (Song et al. 2024). Greater H_v_ reflects a larger potential hydraulic conductivity relative to leaf area, which reduces the xylem tension necessary to supply the evaporative demand from the foliage (Gattmann et al. 2023), thereby sustaining the delivery of water to the guard cells, maintaining their turgor pressure, and allowing stomata to remain open to support maximal g_sw._ It is important to note that slightly higher VPD during recovery in the drought treatments contributed to reduced g_sw_ in addition to H_v_, as both of these factors were related to g_sw_ (Fig. 3). Reduced growth rates during mild drought stress likely led to the formation of fewer or smaller xylem tracheids (Olano et al. 2014), leading to lower hydraulic conductivity per area of new growth than the control. As such, g_sw_ remained below control levels during stress recovery in trees with a similar H_v_ (Fig. 2a). Reduced xylem conductivity per area of growth suggests that previously drought-stressed trees may require a greater stem basal area to achieve the same g_sw_ as unstressed trees, particularly in plants exposed to severe drought which develop embolism. Taken together, these results emphasize how stem anatomical constraints on hydraulic supply strongly regulate gas exchange in conifers (Gattmann et al. 2023; Knüver et al. 2025) and may ultimately limit the recovery of gas exchange following any intensity of drought stress.

### Drought severity increasingly limits recovery of C accumulation

Recovery of C assimilation was limited according to drought severity. During recovery from mild drought stress, photosynthesis gradually increased in-step with g_sw_, suggesting the nonimmediate photosynthesis recovery from mild stress was due to stomatal limitations. In contrast, following severe stress, the recovery of A_net_ was initially decoupled from g_sw_. This occurred despite an immediate increase in A_net,_ but not g_sw_ following rewatering, indicating A_net_ was initially more sensitive to rewetting. However, A_net_ required longer than g_sw_ to reach predrought levels, meaning WUE_i_ remained below control levels during this time period. This suggests that A_net_ was ultimately less sensitive to the recovery of soil water availability than g_sw_ following severe stress. To balance ATP and NADPH regeneration to lower Rubisco demand during drought, plants actively minimize electron flow through photosystems I and II (Pandey et al. 2022; Alongi et al. 2024) and downregulate Rubisco activity and content (Parry et al. 2002). While reduced photosynthesis can occur due to NSC feedback inhibition during periods of reduced sink-activity (Tian et al. 2024), we did not observe evidence of NSC accumulation during recovery. Our observed decoupling of g_sw_ and photosynthesis following stress release indicates that short-term recovery of C assimilation may be less limited by hydraulic constraints than by persistent metabolic inhibition of the photosynthetic apparatus following severe stress. In addition to metabolic downregulation, other mechanisms could explain the observed initial nonstomatal limitation of photosynthesis. Photoinhibition, particularly damage to photosystem II due to the production of reactive oxygen species, may impair recovery until repair processes restore photosystem functionality (Schönbeck et al. 2022). Furthermore, reduced mesophyll conductance can persist independently of g_sw_, thereby increasing the resistance of CO_2_ diffusion (Galle et al. 2010). These alternative mechanisms may act independently or in concert with biochemical downregulation to limit initial photosynthesis recovery following severe drought events.

These drought-severity dependent limitations to C assimilation recovery were clearly reflected in whole-tree C accumulation. While mild-stressed plants accumulated on average 45% less C per day than the control plants throughout the drought period, net C accumulation rates were close to control levels during the recovery period. This occurred despite generally lower A_net_ in mild compared with control trees being compensated for by lower root respiration (Figure S4). In agreement with hypothesis (2), severe drought stress-treated plants accumulated 69% less C per day than the control-treated plants throughout the entire recovery period, with final daily accumulation rates still 51% lower. This largely confirms other work demonstrating strong negative relationship between drought severity and recovery of C accumulation rates (Martínez-Sancho et al. 2022). Moreover, these results emphasize that the recovery potential following droughts of varying severities are directly related to the degree and mechanisms of physiological limitation that occur in response to the stress event (Ruehr et al. 2019).

### Reduced recovery of growth not limited by NSC

Drought-induced reductions in growth persisted through recovery, with 25% less basal growth during recovery from mild stress and 42% less from severe stress. The stronger limitation of growth by severe stress can be partially attributed to a delay in the resumption of growth, as growth was minimal during the first week of recovery, suggesting a temporary delay due to cellular repair (Kannenberg et al. 2022; Lv et al. 2022) or due to regaining phloem functionality (Rehschuh et al. 2020). Our observed growth rate reductions following both mild and severe drought stress are difficult to explain under the sink-driven model of C accumulation. In a sink-driven model of C accumulation, the capacity for growth, as determined mostly by nighttime cell turgor conditions, regulates the amount of C intake via photosynthesis during the day (Körner 2015). While we did not observe differences in branch Ψ_md_ across our stress treatments during recovery, it is possible that small but physiologically relevant differences in Ψ were present but remained below our detection threshold or sampling timeframe.

Reduced growth following either stress treatment could not be attributed to increased C allocation to storage, as NSC concentrations were maintained at or above nominal levels throughout drought and recovery periods. However, it is important to acknowledge that our severe drought treatment was relatively intense, as Ψ_md_ rapidly decreased, leading to individuals having a net negative C balance during the final 8 d of drought on average (Fig. 4b), during which NSC decreased (Fig. 5c). It is likely that a more gradual drought would extend the period of a negative C balance and ultimately lead to NSC depletion below control levels. As total NSC concentrations did not deplete below control levels in either the mild or severe treatment, refilling of NSC did not appear to limit growth recovery as some studies suggest (Kannenberg and Phillips 2020; Guo et al. 2021), in contrast to hypothesis (3). We did observe an increase in stem starch concentration around 2 wk into recovery; however, starch ultimately returned to control levels, representing only a temporary increase in C allocation to storage and likely was a result of reduced growth sink strength.

Nonetheless, we recognize that stem NSC concentrations alone likely oversimplify the available carbon for metabolic and structural demands. Drought events can additionally constrain NSC accessibility by tissue compartmentalization, as well as limitations to transport and remobilization (Hartmann and Trumbore 2016). While stem NSC concentrations remained stable, it is possible that other large NSC pools, such as those in root tissue, remained physiologically inaccessible. Therefore, we conclude that the recovery of stem growth is unlikely to be limited by NSC availability following stress (Palacio et al. 2014) but cannot rule out constrained NSC mobilization as a contributing factor to delayed growth resumption.

### Modified diurnal patterns restrict growth recovery

A further investigation into the timing of growth revealed key diurnal differences between the control and drought treatment plants. Specifically, the higher daily growth rate in control plants appeared to be largely due to daytime growth, which progressively increased throughout the experimental period (Fig. 6b). In contrast, daytime growth was absent in the mild and severe stress treatments during the drought period. Daytime growth appeared in the mild treatment by the third week of recovery but was delayed until the final week of recovery in the severe treatment (Fig. 6b). As with g_sw_, the appearance and degree of daytime growth was strongly related to sapwood development (estimated via H_v_) at the beginning of each day (Figure S5). This was likely due to a greater stem hydraulic supply from increases in sapwood area buffering against daytime changes in xylem tension (Zheng et al. 2022; Ziegler et al. 2024), allowing for growth-favorable turgor conditions to be maintained during the day. This contrasts with the general understanding of tree diurnal growth patterns, where high daytime VPD is expected to restrict growth processes to night (Zweifel et al. 2021). In agreement with this, we found that increasing daytime VPD did negatively affect daytime growth (Figure S5). Interestingly, daytime growth appeared possible once H_v_ reached ∼0.08 under moderate VPD (Figure S5), corresponding to the point in which the seasonal max g_sw_ was reached in both the control and mild stress treatments (Fig. 2a). Daytime growth in the severe treatment, however, did not occur until the final week of recovery (Fig. 6b), corresponding to a notably higher H_v_ (Fig. 2a). This likely was due to the presence of embolism formed during severe drought stress (Fig. 1a), which would reduce stem hydraulic conductance of the existing stem basal area (Rehschuh et al. 2020).

Taken together, our findings suggest that drought-induced modifications to sapwood development largely regulate the capacity for juvenile trees to optimize growth patterns across a greater diurnal scale. With daytime growth constituting up to 30% of daily growth in control trees, drought-induced delays in stem hydraulic development may lead to substantial reductions in seasonal biomass accumulation. Furthermore, these results highlight the importance of the drought timing within the growing season. Drought events occurring early in the growing season, before sufficient sapwood has developed to support and canopy gas exchange, may be particularly disruptive (Ruehr and Nadal-Sala 2025). By limiting the formation of hydraulic capacity needed to sustain photosynthesis and growth, such early-season droughts are likely to lead to prolonged recovery periods and have the potential to carry-over into the next season.

## Conclusion

**Figure 7.**
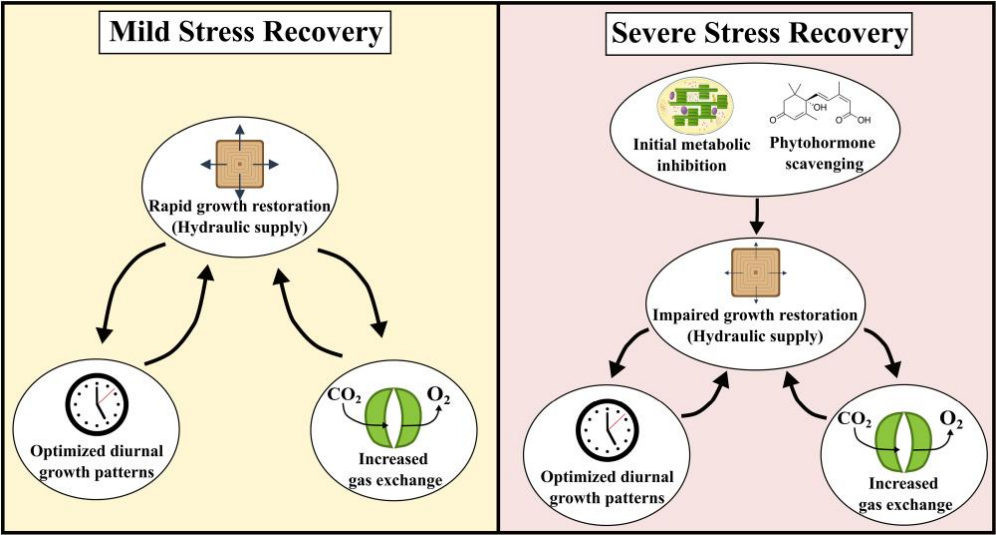
Schematic overview of the mechanistic recovery pathways. Represented are recovery pathways following mild stress (left) and severe stress (right). The restoration of stem hydraulic development positively influenced both the restoration of gas exchange as well as the expansion of diurnal growth patterns, with this pathway substantially delayed following severe stress.

Our findings demonstrate that tree recovery following drought is primarily governed by stem hydraulic constraints, with reduced sapwood development limiting the recovery of both gas exchange and growth in juvenile *P. menziesii* (Fig. 7). While carbon assimilation declined with drought intensity, severe stress induced an initial metabolic inhibition of assimilation, yet recovery was ultimately constrained by limited sapwood development. Similarly, nonstructural carbohydrate reserves in the stem remained abundant, indicating that carbon likely did not limit growth. Instead, reduced sapwood development delayed the seasonal appearance of daytime growth particularly under severe stress, where daytime basal expansion was largely absent. These drought-induced constraints on stem hydraulic development not only slowed gas exchange recovery but also suppressed seasonal carbon accumulation. As this relationship was identified in a singular conifer species with strong stomatal regulation, these mechanisms may not generalize to angiosperms or species with anisohydric hydraulic strategies. Nonetheless, our findings highlight the importance of drought seasonality, as drought events occurring earlier in the year, before sufficient sapwood area has developed to sustain the newly developed leaf area, may disproportionately affect gas exchange and growth and ultimately lead to extended recovery periods. Together, these results provide a process-based explanation for the observance of prolonged reductions in forest productivity following drought events.

## Materials and methods

### Plant material and environmental conditions

We obtained 72 bare-root 3-yr-old *P. menziesii* seedlings (HkG 85305) from a tree nursery in March 2023 (Forstbaumschulen Gracklauer, Gunzenhausen, Germany). Seedlings were placed in individual 5.7 L pots containing an inorganic substrate mixture of fine quartz sand (0.1 to 1.2 mm), medium grain sand (1 to 2.5 mm), perlite, and vermiculite (2:2:2:1), with 12 g extended-release fertilizer and 2 g micronutrient mix (Osmocote 5 8 to 9 M, Micromax Premium; ICL Specialty Fertilizers, Geldermalsen, Netherlands).

In May 2023, seedlings were moved into an experimental greenhouse facility in Garmisch-Partenkirchen, Germany (708 m a.s.l., 47°28′32.9″ N, 11°3′44.2″ E) to acclimate for 4 wk.) to acclimate for 4 wk. Seedlings were automatically drip irrigated with 150 mL water twice daily (07:00, 21:00; Rain Bird, Azusa, United States), with LED grow lamps maintaining a 15 h photoperiod (LED-KE 400 VSP, DHLicht, Wuelfrath, Germany). Throughout the acclimation and experimental period, continuously measured photosynthetic active radiation (PAR; PQS 1, Kipp & Zonen, Delft, The Netherlands) reached daytime averages of 600 µmol m^−2^ while air temperature and relative humidity were maintained at 23 ^°^C and 60%, respectively (see Fig. S7 for greenhouse growth conditions).

### Experimental conditions

The seedlings were randomly assigned to either a well-watered control (*n* = 24), mild-drought (*n* = 24), or severe-drought (*n* = 24) treatment. Six seedlings from each treatment were placed in custom-built tree gas flux chambers (referred to as chamber seedlings); however, 3 individuals were ultimately removed due to exceptionally high initial transpiration rates (outliers), resulting in a sample size of *n* = 6 for control, *n* = 5 for mild, and *n* = 4 for severe treatments. The remaining seedlings were placed on separate benches by treatment (referred to as pilot seedlings) and were utilized for destructive samples as to not influence gas-exchange measurements.

On 6 June 2023 following leaf out, irrigation was withheld completely from the severe-drought treatment (0 mL daily), reduced in the mild-drought treatment (60 mL daily), while the control treatment continued to receive drip irrigation (300 mL daily). SWC was measured throughout the experimental period in all gas flux chamber pots, and in 4 pilot pots per treatment (10HS, Meter Group, United States), and informed drip-irrigation modifications to minimize variation within and between the pilot and corresponding chamber treatments. Targeted soil volumetric water content (SWC) by the end of drought were 0% in severe, 10% in mild, and >20% in the control. SWC progressively decreased throughout the drought period in the noncontrol treatments. The mild drought treatment attained the targeted 10% SWC halfway through drought stress and was maintained, reaching 10.3 ± 1.3% in chamber seedlings and 9.6 ± 0.7% in pilot seedlings by the end of drought stress. The severe drought treatment continuously dehydrated throughout the drought period, with chamber and pilot seedlings reaching values close to zero by the end of drought stress.

All gas flux chamber pots were equipped with dendrometers installed ∼5 cm above the soil to continuously measure relative changes in diameter (DD-S, Ecomatik, Germany, 1.5 µm resolution). Absolute diameters were calculated in comparison to the initial diameter measured by a caliper and were transformed into basal area. These measurements of basal area were used to infer sapwood area, which we utilized to calculate the Huber value (H_v_, estimated as the sapwood area to leaf area ratio).

Growth was determined based on the zero growth concept (Zweifel et al. 2016), which assumes no growth during periods of stem dehydration when the stem diameter is below the previously recorded maximum (Ziegler et al. 2024). Growth data during recovery were reported starting the second day of recovery to account for immediate stem rehydration. Midday branch water potential (Ψ_md_) was frequently measured on axial branches of randomly selected pilot seedlings (*n* = 3 to 5 per treatment) using a Scholander-type pressure chamber (Model 1505D, PMS Instruments, Oregon, United States). Midday branch water potential was selected to identify the highest daily xylem tension experienced during treatment progression. An end-drought target Ψ_md_ of ∼−4 MPa in the severe drought treatment was chosen to induce hydraulic damage but not mortality, corresponding to a percent loss of conductivity of 70% to 85% (Chauvin et al. 2019).

On 4 July 2023, mild and severe drought treatment irrigation returned to 300 mL daily, while all parameters continued to be measured until the end of recovery on 9 August 2023. Following release of drought stress, mild and severe drought treatment SWC quickly increased to control values (>20%) and were maintained throughout the recovery period (Fig. S8).

**Figure 8.**
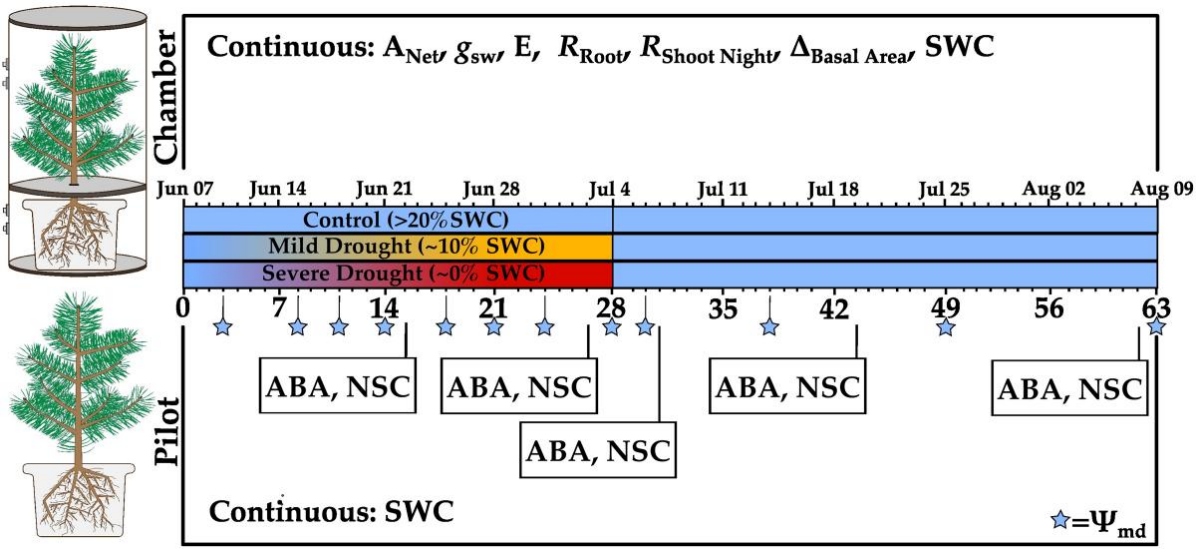
Experimental design overview and sampling timeline, indicated by both calendar date (upper timeline) and experimental day (lower timeline). Seedlings were divided between custom-built gas exchange chambers (upper left, *n* = 4 to 6 per treatment) and pilot seedlings for destructive harvest (lower left, *n* = 17 to 18 per treatment). Chamber seedlings were measured hourly for net photosynthesis (A_Net_), stomatal conductance (g_sw_), transpiration (E), root respiration (R_Root_), shoot respiration (R_Shoot_, nighttime only), with continuous measurements of stem basal area (Δ_Basal Area_) and volumetric SWC. Pilot seedlings were destructively harvested at the indicated timepoints for midday branch water potential (Ψ_md_), foliar abscisic acid (ABA), and branch NSC, with SWC being continuously measured (*n* = 3 to 4 per treatment).

Following the end of the recovery period, all chamber seedlings were harvested for biomass and separated into leaf, aboveground woody tissue (stem + branch), and belowground woody tissue (root). Root:shoot is defined as the ratio of belowground biomass (root) to aboveground biomass (leaf + stem + branch). Biomass was then dried for 72 h at 60 °C, with dry weight recorded thereafter. Total leaf biomass was then used to calculate leaf area for gas exchange standardization. Leaf area was measured on a subset of fresh leaves, with those leaves subsequently dried to determine the leaf area to dry weight ratio, which was then used to transform total leaf biomass into leaf area. We assume that leaf area for each individual was constant throughout the duration of the experiment, as leaf-out and needle elongation was complete at the onset of gas-exchange measurements. Minor leaf shedding and lammas growth occurred on a few individuals under drought treatment; however, this growth was visually estimated to contribute very little to the total leaf area (<5%). An experimental overview including the measurement timeline is available in Fig. 8.

### Tree gas flux chamber system

Gas exchange (H_2_O and CO_2_) was continuously measured in above- (shoot) and belowground (root) compartments using a custom-built tree gas flux chamber system (for details see Birami et al. 2020 and Rehschuh et al. 2022). Chambers were continuously supplied predefined 445.6 ± 0.2 ppm CO_2_ and 8.0 ± 0.0 mmol H_2_O concentration air, with both the provided airstream (reference) and returned airstream (sample) measured for absolute and differential CO_2_ and H_2_O (Li-840, Li-7000; Li-Cor, Lincoln, Nebraska, United States). CO_2_ was supplied above ambient levels to partially offset reductions in individual chamber CO_2_ which occur during photosynthesis. Additionally, 2 empty chambers containing the same C-free potting substrate were utilized to account for background fluxes and were accordingly subtracted from their respective sample chamber sections. We note that these offsets were relatively minor across chamber sections (+0.15 ± 0.09 ppm CO_2_ and 0.03 ± 0.11 mmol H_2_O on average). LI-COR devices were calibrated every 2 wk using zero CO_2_ and H_2_O air. Air temperature (T_air_) of shoot compartments was regulated by fast-response thermocouples (5SC-TTTI-36-2M, Newport Electronics GmbH, Deckenpfronn, Germany). Air measurements were at 10 s intervals, with 1 measurement cycle through all chambers lasting ∼80 min. For environmental conditions in the gas exchange chambers, see Fig. S1. Net photosynthetic assimilation per leaf area (*A*_net_), root respiration (*R*_root_), stomatal conductance per leaf area (g_sw_), and transpiration per leaf area (*E*) were calculated as in Rehschuh and Ruehr (2022). Carbon fluxes were calculated separately for each chamber compartment (root and shoot) without normalizing by leaf area in order to capture the whole-tree carbon budget.

Molar CO₂ fluxes for each chamber compartment were derived from the difference in dry-air CO₂ mole fractions between sample and reference air streams:


(1) $$F_{CO_{2},comp}\;=\;-{\dot{m}}_{comp}(C_{sample,comp}-C_{reference,comp})$$

where:

$F_{CO_{2},comp}\;$ : molar CO_2_ flux of the chamber compartment *comp* (mol CO_2_ s^−1^)

${\dot{m}}_{comp}$ : molar flow rate through the chamber compartment (mol s^−1^)

$C_{sample,comp}$ : CO_2_ mole fraction in the chamber sample air after correction for water dilution (mol CO_2_ mol^−1^ dry air)

$C_{reference,comp}$ : CO_2_ mole fraction in the reference airstream after correction for water dilution (mol CO_2_ mol^−1^ dry air)

Daily net carbon exchange for each individual ($C_{daily})$ was then obtained by integrating average molar fluxes (mol CO_2_ s^−1^) from each compartment throughout the day and then multiplying by the molar mass of C and seconds in a day:


(2) $$C_{daily}=(\bar{F_{CO_{2},shoot}}+\bar{F_{CO_{2},root}})\;*\;M_{C}\;*\;s_{day}$$

where:

$C_{daily}$ : daily net C exchange for the study individual (g C day^−1^)

$\bar{F_{CO_{2},shoot}}$ and $\bar{F_{CO_{2},root}}$: daily average molar CO_2_ flux (mol CO_2_ s^−1^) from the shoot and root compartments, respectively

$M_{C}$ = 12.01 g mol^−1^: molar mass of carbon

$s_{day}$ : 86,400 s day^−1^

Cumulative carbon accumulation over the course of the experiment was then calculated as the sum of daily values:


(3) $$Plant\;C(d)=\overset{d}{\underset{i=1}{\sum }}\;C_{daily,i}$$

where:

PlantC(d) : cumulative carbon accumulation up to day *d* (g C)

$C_{daily,i}$ : net daily carbon accumulation on day *i* (g C day^−1^)

d: day index (1 ≤ d ≤ total number of days in the experiment)

### Nonstructural carbohydrate quantification

Axial stems (∼8 cm) were harvested from randomly selected pilot trees of all treatments (*n* = 5 to 7) and microwaved for 3 60 s increments to halt metabolic activity. Tissues were then dried for 72 h at 60 °C and ground to a fine powder. Stems were analyzed for starch and sugar (sucrose + fructose + glucose) concentration using a standardized enzymatic method (Landhäusser et al. 2018). Free sugar concentrations were determined after conversion to glucose-6-P via invertase and isomerase. Starch concentration was determined by hydrolyzing α-amylase and amyloglucosidase to convert starch to glucose-6-P. Dehydrogenase was used to oxidize glucose-6-P to gluconate-6-P and absorbance was read at 340 nm on a 96-well microplate photometer (Epoch 2, Agilent, Santa Clara, California, United States). Total NSC is considered the sum of free sugar and starch, with all NSC reported in % dry weight.

### Abscisic acid quantification

Needles were collected from the harvested axial stems and immediately frozen in liquid nitrogen until ABA quantification at the destructive timepoints (*n* = 5 to 7/treatment). Foliar ABA levels were determined via physicochemical methods (McAdam and Brodribb 2014), where samples were fully homogenized and 15 ng of [^2^H_6_]ABA internal standard was added to each sample. Endogenous ABA was extracted from the homogenized foliar tissue, with an aliquot taken and dried under vacuum until completion. Samples were then resuspended in 200 μL of 2% acetic acid, with ABA levels then quantified using liquid chromatography–mass spectrometry (Agilent 6400 LC/MS, United States).

### Statistical methods

Statistical analysis was conducted in the R statistical programming environment v4.3.2 (R Core Team 2022). Linear fixed-effect models were used to identify fixed treatment differences for all measured parameters at single timepoints (Ψ_md_, ABA, NSC, biomass metrics, gas exchange, as well as cumulative C uptake and growth for the drought, recovery, and total experiment timeframes). For timeseries regression analysis (daytime growth and final C accumulation rates), mixed-effect models were used to account for repeated measures by including individuals as a random effect, while treatment remained as a fixed effect (*lme4* package, Bates et al. 2015). Exploratory mixed-effect models describing g_sw_ and daytime growth throughout the experimental period were fit using an interaction term between VPD and H_v_ with treatment as an additive effect, while including individuals as a random effect. All models were analyzed via diagnostic plots to verify parametric modeling assumptions of normality, equal variance, and influential points, with log-response transformations used for data which violated assumptions. Post hoc differences between treatments were determined using Tukey's Honest Significant Distance with the Kenward–Roger degree of freedom method with a 95% confidence interval and a 0.05 significance level (*emmeans* package; Lenth et al. 2024). Standard error is reported for all included measurements. For daytime (09.00 to 14.00) and nighttime (00.00 to 05.00) averages, parameters were first averaged for each individual, with standard error then calculated per treatment.

## Supplementary Material

kiaf654_Supplementary_Data

## Data Availability

Gas exchange data is available through PANGAEA Data Publisher for Earth & Environmental Science (https://doi.org/10.1594/PANGAEA.988300). All other data used for analysis and visualization are available upon request.

## Supplementary material

The following materials are available in the online version of this article:

Supplementary Figure 1. Environmental conditions in the tree flux chambers.

Supplementary Figure 2. Timeseries of transpiration data.

Supplementary Figure 3. Above and belowground net carbon accumulation.

Supplementary Figure 4. Root and nighttime shoot respiration timeseries.

Supplementary Figure 5. Effect plot of exploratory model evaluating stem hydraulic supply.

Supplementary Figure 6. Root:shoot ratio at the end of the experimental period.

Supplementary Figure 7. Greenhouse environmental parameters.

Supplementary Figure 8. Soil water content during treatment progression.

Supplementary Table 1. Correlations between gas exchange and stem hydraulic supply.

Supplementary Table 2. Measurements of biomass at the end of the experimental period.

## Dive Curated Terms

The following phenotypic, genotypic, and functional terms are of significance to the work described in this paper:

ICL Gramene: AT3G21720
ICL Araport: AT3G21720

carbon CHEBI: CHEBI:27594

starch CHEBI: CHEBI:28017

starch concentration Planteome: TO:0000702

stomatal conductance Planteome: TO:0000522

branch AmiGo: PO:0025073

leaf AmiGo: PO:0025034

NADPH CHEBI: CHEBI:16474
