# Supplementary material for: Drought-induced delays in stem hydraulic development shape gas exchange and growth recovery in Douglas fir
Source: Plant Physiol. 2025 Dec 17;200(3):kiaf654. doi: 10.1093/plphys/kiaf654 (PMC13010329; doi:10.1093/plphys/kiaf654)
Supplement: kiaf654_Supplementary_Data [file kiaf654_supplementary_data.pdf]

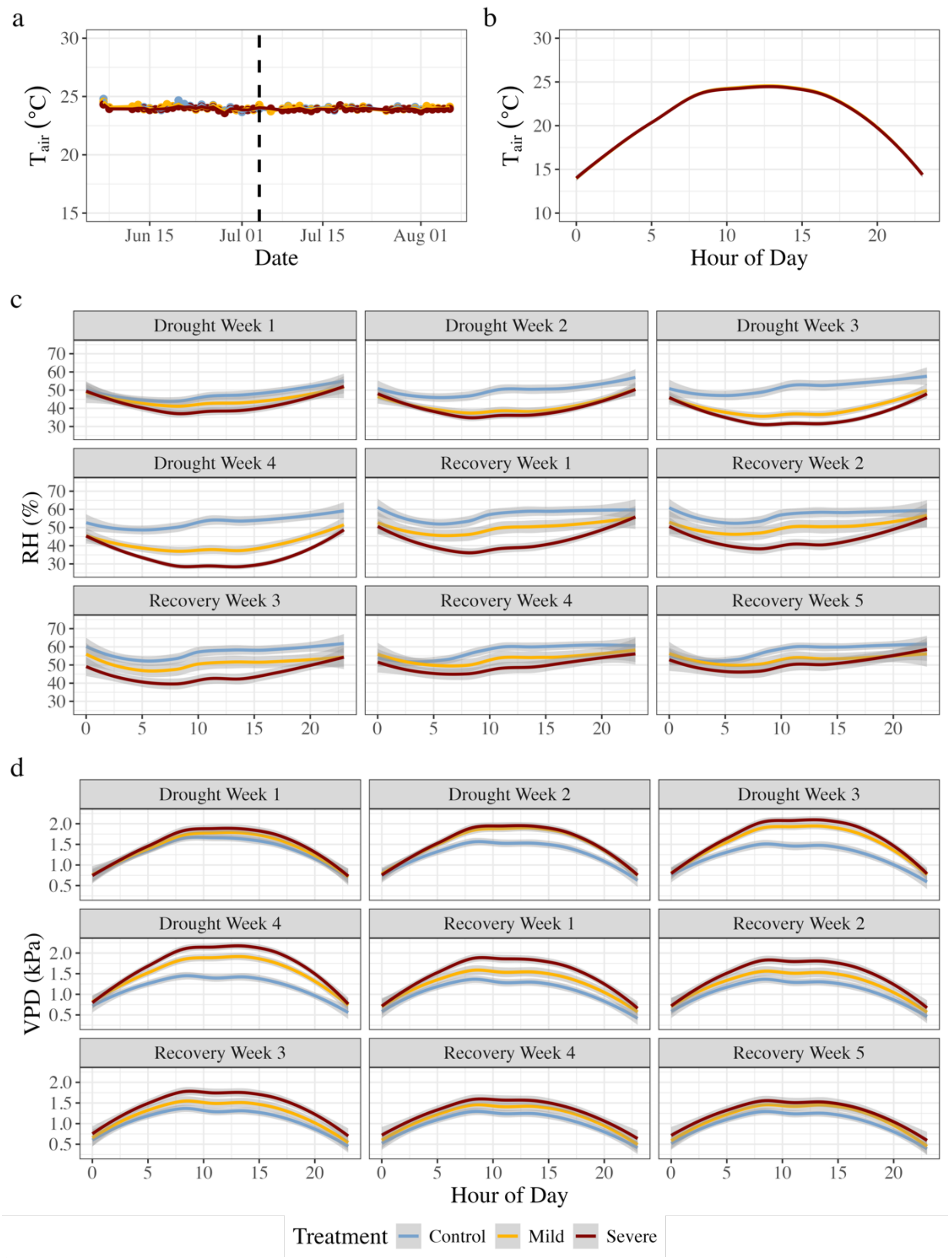

Figure S1

Environmental conditions for chamber seedlings during the experimental period. Reported are mean daytime (09.00 – 14.00) air temperature (T_{air}, a), and the diurnal cycle of air temperature (b) throughout the experiment. Due to drought-induced changes in transpiration, relative humidity (RH, c) and therefore vapor pressure deficit (VPD, d) varied between chamber treatments with drought development, and therefore are displayed for each week in the experimental period. All diurnal cycles are shown with standard error. Note, while photosynthetic active radiation is not reported for chamber seedlings, this can be assumed to be equivalent to the greenhouse PAR shown in Figure S1.

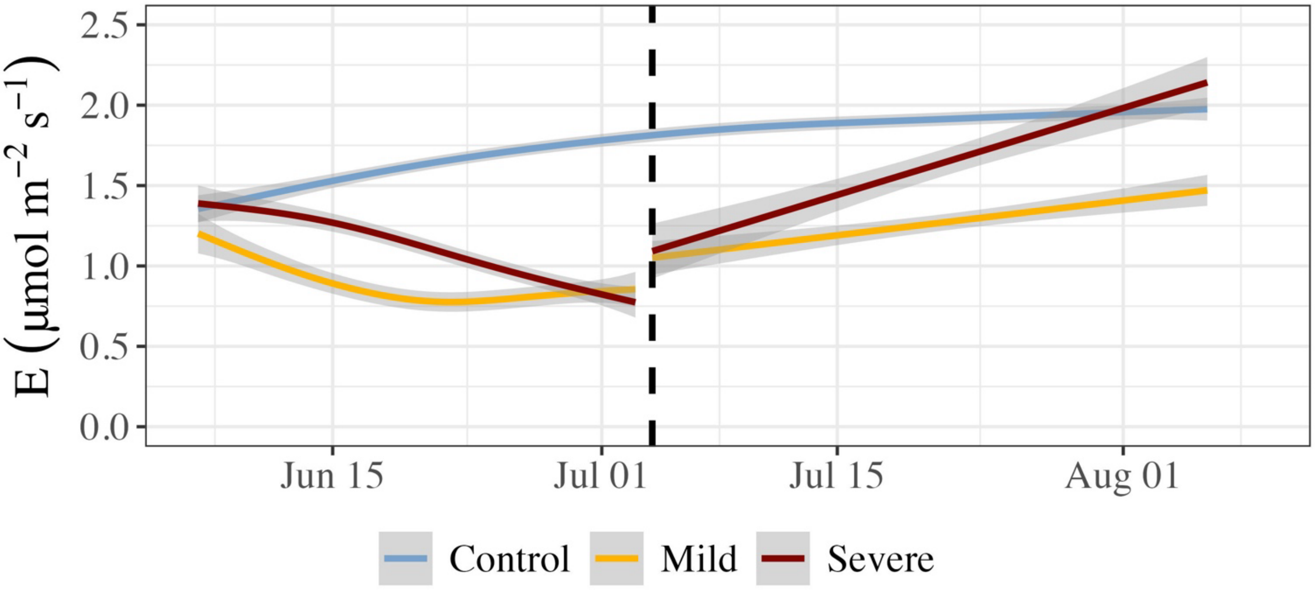

Figure S2
Time series of daytime (09.00 – 14.00 transpiration (E) throughout the experimental period (*n* = 4-6 per treatment). Generalized additive models were fit to produce smoothing lines, with the gray shaded area representing ±SE. The dashed black line indicates the transition from drought to recovery.

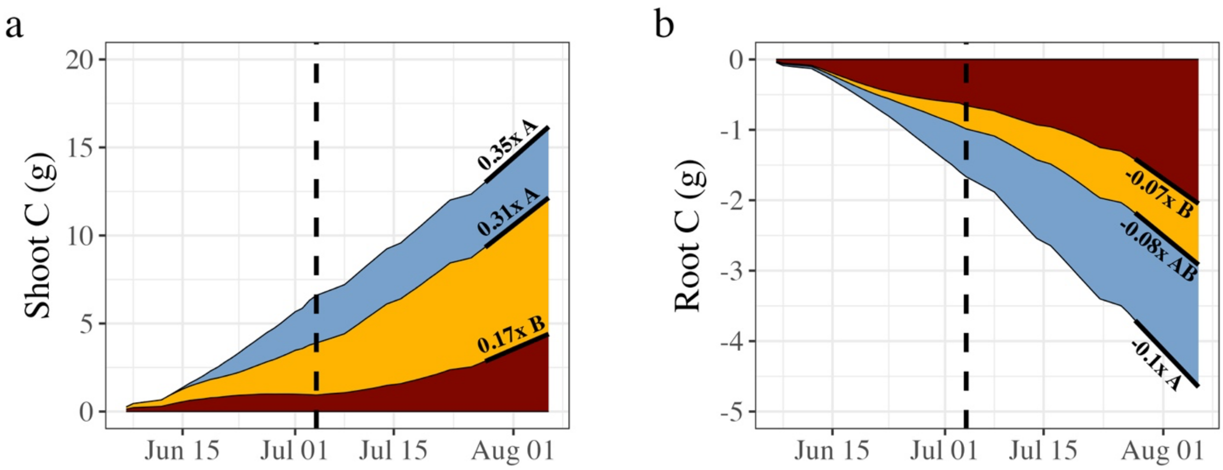

Figure S3

Carbon accumulation is reported in grams of carbon from Douglas fir seedlings in gas exchange chambers. Reported are daily net C accumulation from shoot chamber sections (a) and daily net respiration from root chamber sections (b) over the course of the experiment (*n* = 4-6). The dashed black line indicates the transition from drought to recovery. Linear models are fit for the final 10 days of recovery for each treatment (black lines) with the slope reported to indicate daily C accumulation rates. Significant differences of the slopes (uppercase letters) were calculated using Tukey's Honest Significant Difference.

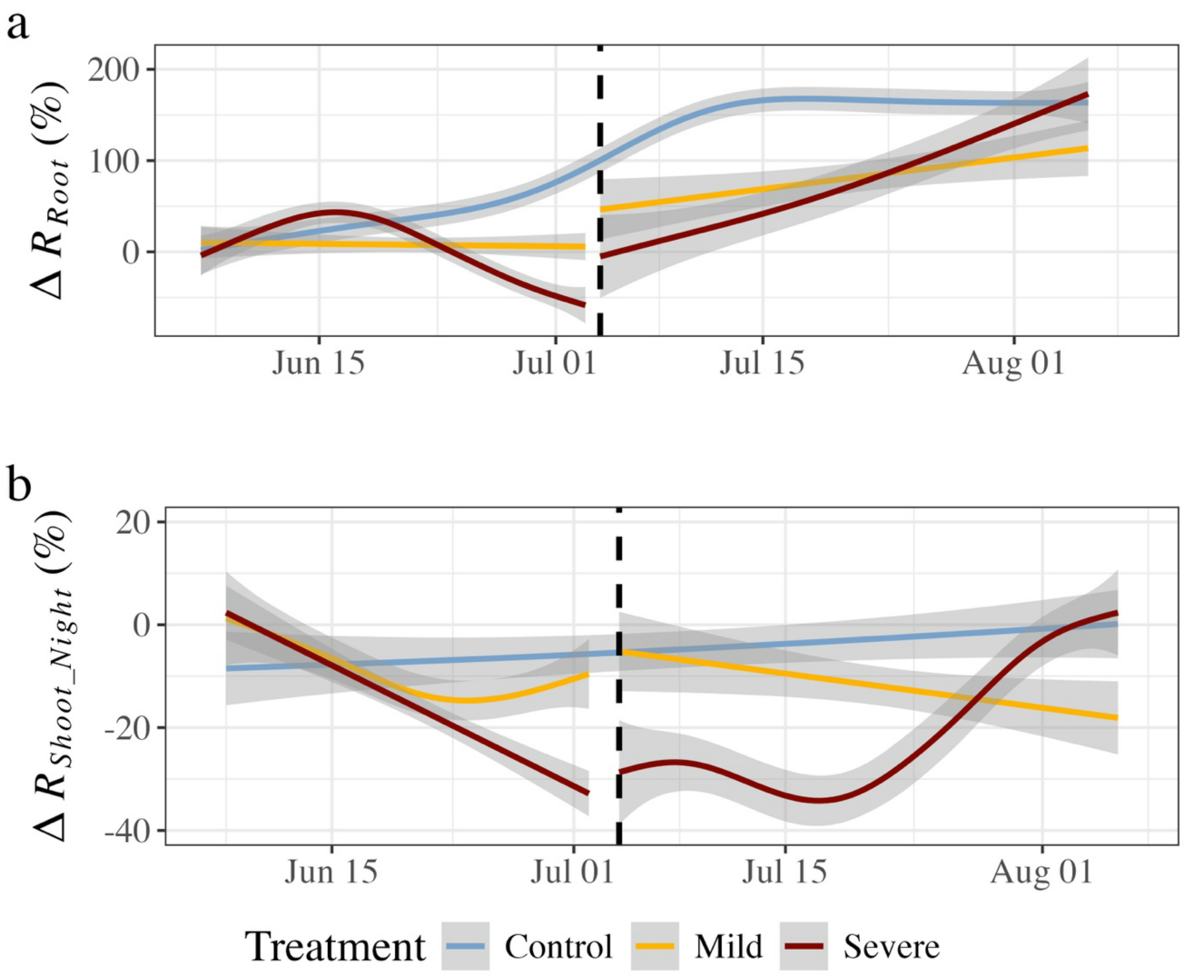

Figure S4
Change in Douglas fir seedling respiration throughout the experimental period (drought and recovery). Due to growth of woody tissues, data is standardized to percent change from the experimental start rather than biomass. Generalized additive models were fit on individual daily mean belowground respiration rates (R_{Root}, a), as well as nighttime aboveground respiration (R_{Shoot_Night}, b). The gray area represents ±SE (n = 4-6) while the dashed black line in both panels indicates the transition from drought to recovery.

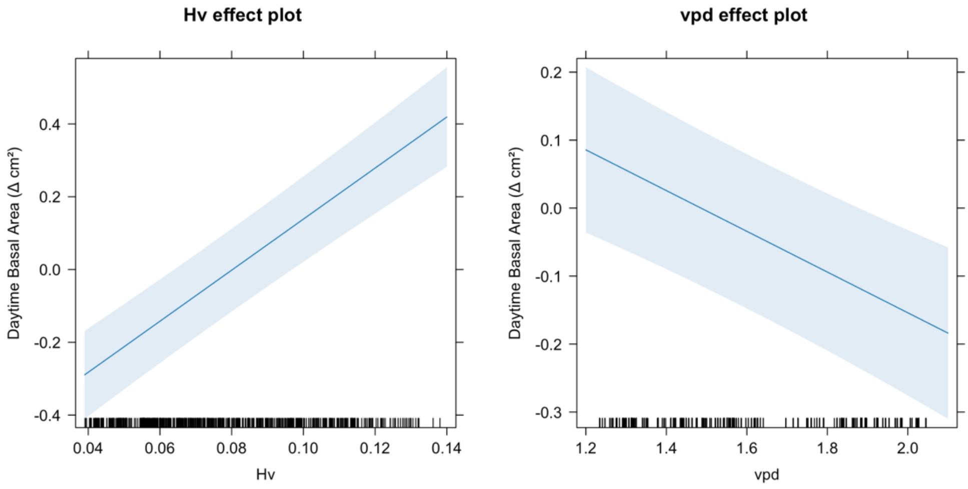

Figure S5

Effect plots from an exploratory model evaluating the effects of stem hydraulic supply at the start of the day (Huber value; H_v) and vapor pressure deficit (vpd) on daytime change in basal area, calculated as the difference in basal area between 5.00 and 17.00. The model did not reveal an interaction between H_v and vpd, with no additional variation explained by treatment.

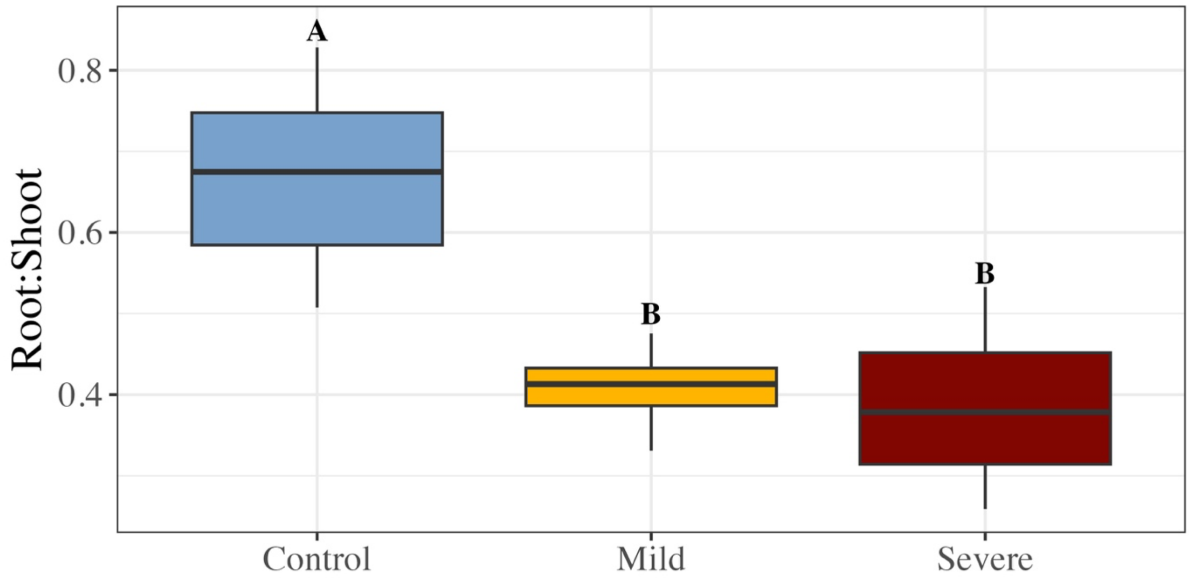

Figure S6
The root:shoot ratio is reported for all seedlings utilized in the gas exchange chambers at the end of the experiment (c, n = 4-6 per treatment). Uppercase letters indicate significant pairwise differences determined post-hoc using Tukey's Honest Significant Difference. The shaded area of the boxplot represents the interquartile range, with the whiskers representing 1.5x the interquartile range, while the solid line representing the treatment median value.

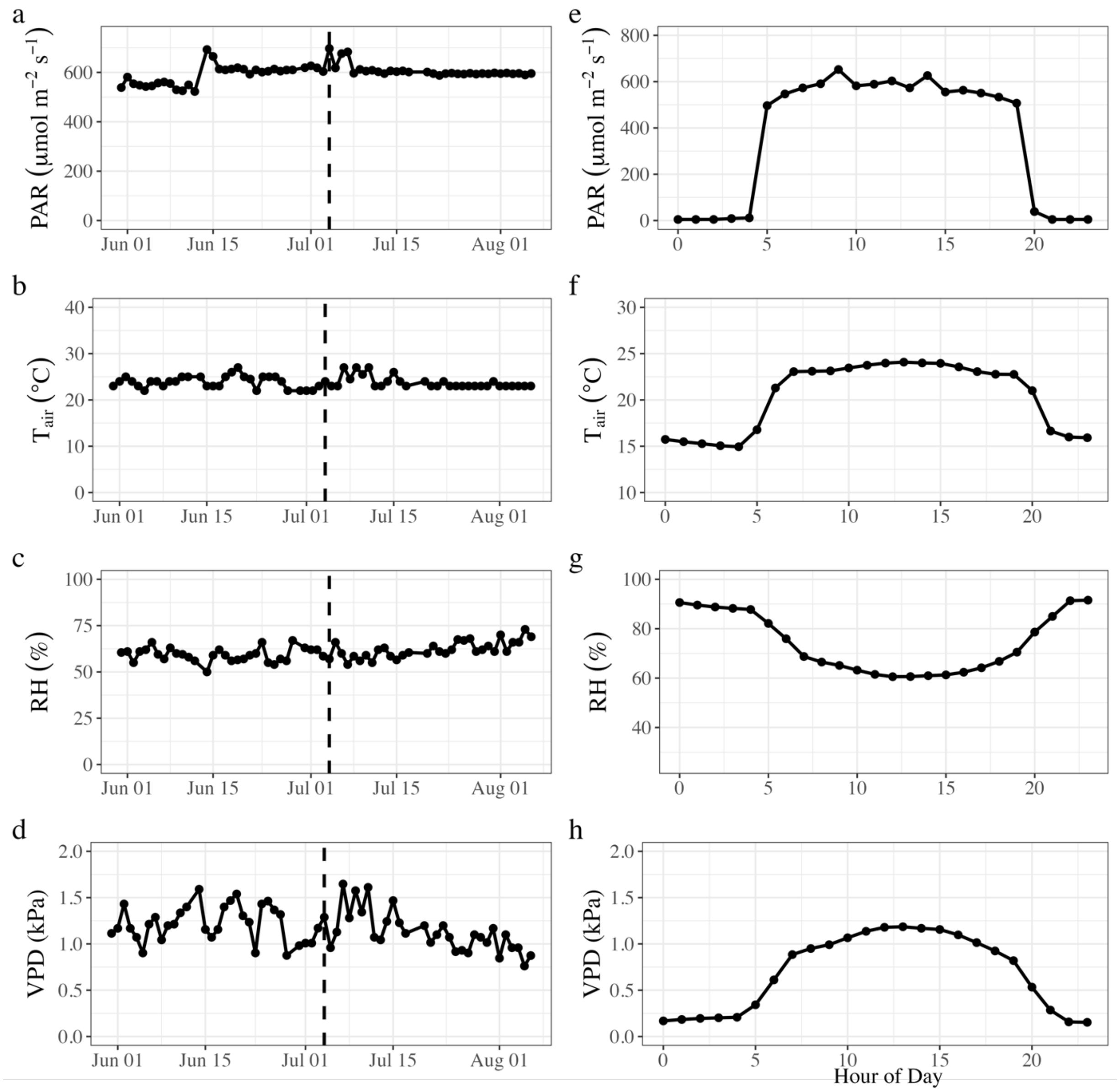

Figure S7

Time series visualization of greenhouse growth conditions. Displayed are the mean daytime (09.00 – 14.00) greenhouse photosynthetic active radiation (PAR, a), air temperature (T_{air}, b), relative humidity (RH, **c**), and vapor pressure deficit (VPD, d) during the experimental drought and subsequent recovery period for pilot seedlings. The dashed black line indicates the transition from drought to recovery. Diurnal cycles of the environmental conditions are reported (e-h).

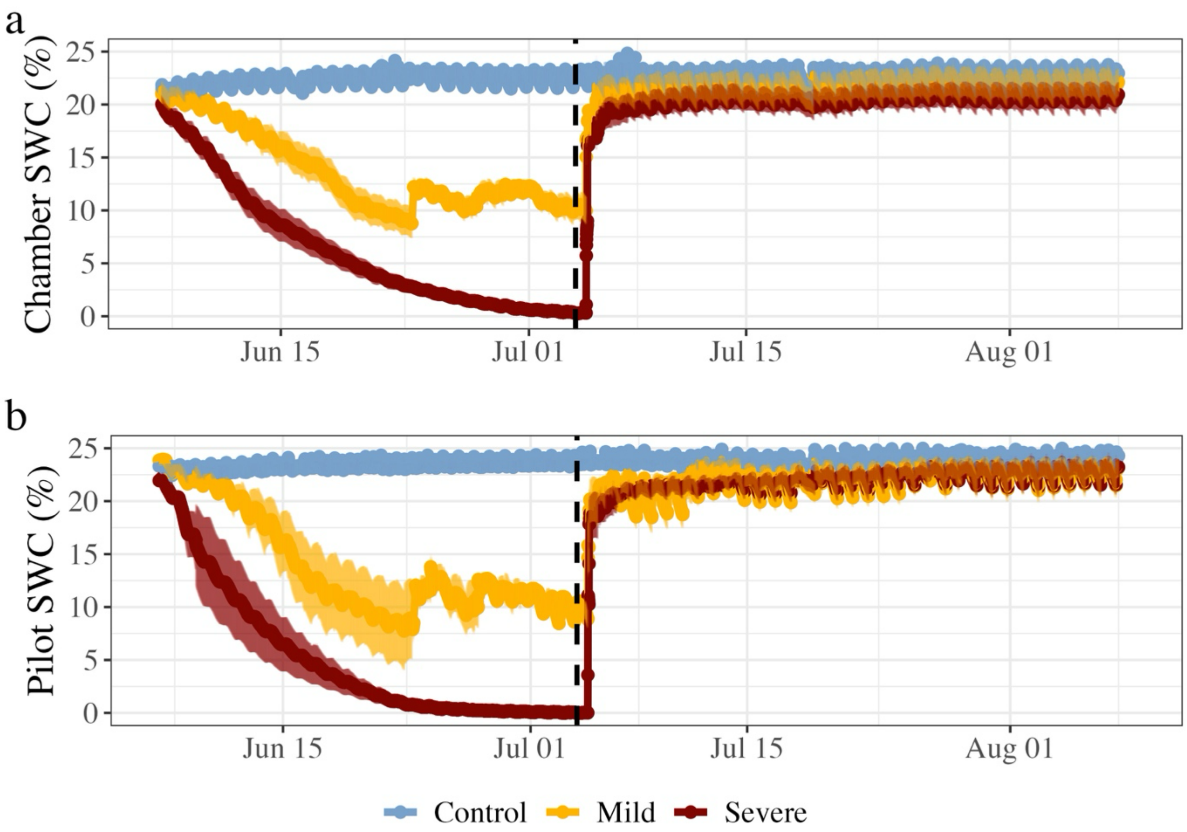

Figure S8
Soil water limitation imposed on seedlings by drought treatments (Control, Mild, Severe) throughout the experiment. Volumetric soil water content (SWC) is shown for gas exchange chamber seedlings (a, *n* = 4-6 per treatment) and pilot seedlings (b, *n* = 3-4 per treatment). Shaded area represents ±SE for each timepoint, while the dashed black line indicates the transition from drought to recovery.

Table S1

Correlation matrices of average daytime (09.00 – 14.00) net photosynthesis (A_{Net}), stomatal conductance (g_{sw}), and the stem sapwood area : leaf area ratio (H_v) for each treatment throughout the course of the experiment. We computed Pearson correlations after adjusting for the effect of vapor pressure deficit and repeated measures using linear mixed-effects models. Correlation matrices with associated p-values were generated separately for each treatment group.

Treatment	Variable	A_{Net}	g_{sw}	H_v
Control	A_{Net}	1.00 (—)	0.32 (p<0.001)	0.42 (p<0.001)
	g_{sw}	0.32 (p<0.001)	1.00 (—)	0.18 (p=0.001)
	H_v	0.42 (p<0.001)	0.18 (p=0.001)	1.00 (—)
Mild	A_{Net}	1.00 (—)	0.66 (p<0.001)	0.56 (p<0.001)
	g_{sw}	0.66 (p<0.001)	1.00 (—)	0.59 (p<0.001)
	H_v	0.56 (p<0.001)	0.59 (p<0.001)	1.00 (—)
Severe	A_{Net}	1.00 (—)	0.51 (p<0.001)	0.37 (p<0.001)
	g_{sw}	0.51 (p<0.001)	1.00 (—)	0.51 (p<0.001)
	H_v	0.37 (p<0.001)	0.51 (p<0.001)	1.00 (—)

Table S2
Biomass (g DW) measured during destructive harvest at the end of the experimental period. Reported are tissue means across treatment with standard error. Bold letters indicate significant differences calculated using TukeyHSD.

Treatment	Total biomass	Belowground woody biomass	Aboveground woody biomass	Needle
Control	49.2 ± 7.4 **A**	19.8 ± 2.9 **A**	20.1 ± 2.3 **A**	9.2 ± 0.7 **A**
Mild	45.5 ± 10.5 **AB**	13.1 ± 3.0 **AB**	20.5 ± 4.7 **AB**	11.8 ± 3.2 **A**
Severe	24.8 ± 11.8 **B**	7.1 ± 1.6 **B**	10.8 ± 1.6 **B**	7.0 ± 0.7 **A**
